# Antithrombocytopenic and immunomodulatory potential of metabolically characterized aqueous extract of *Carica papaya* leaves

**DOI:** 10.1080/13880209.2017.1346690

**Published:** 2017-08-24

**Authors:** Varisha Anjum, Poonam Arora, Shahid Husain Ansari, Abul Kalam Najmi, Sayeed Ahmad

**Affiliations:** a Department of Pharmacognosy & Phytochemistry, Faculty of Pharmacy, Jamia Hamdard University, New Delhi, India;; b Department of Pharmacology, Faculty of Pharmacy, Jamia Hamdard University, New Delhi, India

**Keywords:** Dengue fever, thrombocytopenia, immunomodulation, UPLC-qTOF/MS

## Abstract

**Context:***Carica papaya* Linn. (Caricaceae) leaf (CPL) juice has long been traditionally used in ethnomedicine for dengue fever.

**Objective:** The study examines the effects of standardized CPL aqueous extract (SCPLE) on platelet count, extramedullary haematopoiesis (EMH), and immunomodulation in cyclophosphamide (CP)-induced animal model of thrombocytopenia.

**Materials and methods:** The extract was analyzed for myricetin, caffeic acid, *trans*-ferulic acid, and kaempferol using HPTLC for standardization followed by UPLC-qTOF/MS fingerprinting for metabolite signature. The effects of SCPLE (50 and 150 mg/kg p.o.) on proliferative response of platelet count and total leucocyte count (TLC) were observed up to 14 days in Wistar rat. However, delayed-type hypersensitivity (DTH), haemagglutination titre (HT), and *in vivo* carbon clearance were examined as immunomodulatory parameters in albino mice at 150 mg/kg p.o. against CP.

**Results:** The quantitative HPTLC estimation of SCPLE showed the presence of myricetin, caffeic acid, *trans*-ferulic acid, and kaempferol up to 280.16 ± 5.99, 370.18 ± 6.27, 1110.86 ± 2.97, and 160.53 ± 2.48 (μg/g), respectively. Twenty-four metabolites were identified using UPLC-qTOF/MS. Oral administration of SCPLE (150 mg/kg) in thrombocytopenic rats exhibited significant (*p* < 0.01) increase in thrombocytes (1014.83 × 10^3^ cells/mm^3^), DTH response (0.16 ± 0.004), and phagocytic index (63.15% increase) as compared to CP-induced thrombocytopenia group. Histopathological studies showed minimal fibrosis in spleen histology.

**Discussion and conclusions:** Results suggest CPL can mediate the release of platelets providing the means for the treatment and prevention of dengue.

## Introduction

**Figure 1.**
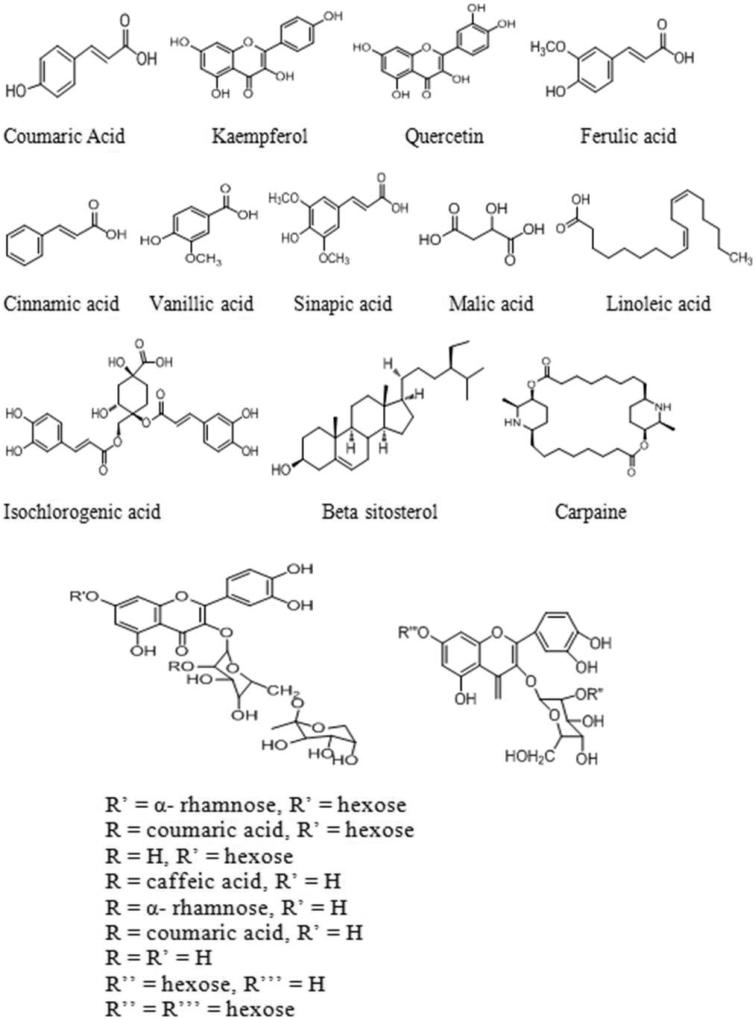
Proposed chemical structures of tentatively identified molecules in papaya leaf aqueous extract.

Thrombocytopenia is a condition associated with a lower production of platelets than the normal numbers in the bone marrow and is often multifactorial (WHO 1997). The physiological range for thrombocytes in normal healthy human is 150–400 × 10^9^/L of blood. Thrombocytopenia causes few signs or symptoms with platelet counts in the range of 50–100 × 10^9^/L that represent moderate-to-severe thrombocytopenia. Diseases such as dengue, idiopathic thrombocytopenic purpura, hypersplenism, aplastic anaemia, chikungunya, and drug-induced thrombocytopenia result in a low thrombocyte count in blood (Aster and Bougie 2007). The available methods for treating thrombocytopenia mainly depend on the disease severity (Azarkan et al. 2003). However, a viral disease such as dengue is the most emerging disease and has become a major health concern in recent years. It destroys the immune system and causes headache, inflammation, hypertension, mental disorders, and thrombocytopenia. The death occurs due to its adverse effects on liver and excessive bleeding. Currently, no vaccine is available for dengue and hence common people use papaya leaves to cure dengue as it has tendency to increase the platelet count and showed hepatoprotection against its devastating virus (Ahmad et al. 2011). *Carica papaya* Linn. (Caricaceae) leaf has been used in folk medicine since centuries for wound healing and in treatment of blood disorders, jaundice, and malaria (Bhatt et al. 2001). The papaya leaves contain many active components such as α-tocopherol, ascorbic acid, phenolic acids (*trans*-ferulic acid, *para*-coumaric acid, caffeic acid, vanillic acid), flavonoids (myricetin, kaempferol, quercetin), cyanogenic glycosides, and glucosinolates (Seigler et al. 2002). Recent studies have shown its beneficial effect as an anti-inflammatory (Owoyele et al. 2008), antitumour, immunomodulatory (Otsuki et al. 2010), antioxidant (Imaga et al. 2010), and for wound healing (Gurung and Skalko-Basnet 2009). An increase in platelet and white blood cells (WBC) count within 24 h has been proven clinically with papaya leaf juice (Ahmad et al. 2011; Hettige 2008). Zunjar et al. (2016); in a recent study, it showed increase in platelet count by carpaine obtained from papaya leaf. The present study was performed to validate the SCPLE by high-performance thin layer chromatography (HPTLC) and to examine its platelet increasing effect after oral administration in both normal and CP-induced thrombocytopenic rats, since there are no reports for simultaneous quantification of myricetin, caffeic acid, *trans*-ferulic acid, kaempferol (Figure 1) in papaya leaf. There are few reports available on quantification of phenolic acids and flavonoids using HPTLC such as caffeic acid (Rajani et al. 2001), kaempferol (Ramsewak et al. 2001), as well as for caffeic acid, ferulic acid, and others (Bhandari et al. 2006; Mishra et al. 2005) using visualizing reagents on cyanopropyl and cellulose plates but neither on silica and nor simultaneously developed. The proposed method is relatively simple, simultaneously developed on silica without derivatizing agents, which increases its sensitivity and repeatability and makes the method more economic.

## Materials and methods

### Chemicals and solvents

Formic acid, acetonitrile, methanol, and anhydrous sodium sulphate were purchased from J.T. Baker (Baker Mallinckrodt, Mexico), while diethyl ether, methyl *tert*-butyl ether (MTBE), β-carotene (97%), gallic acid and rutin (94%) were procured from Sigma-Aldrich (St. Louis, MO). The HPTLC plates Si 60 F_254_ (10 × 20 cm) were purchased from E. Merck (Darmstadt, Germany). The myricetin (98%), *trans*-ferulic acid (99%), caffeic acid (98%), and kaempferol (98%) were purchased from Chromadex (Life Technology, Bangalore, India). Solvents used for chromatography were of HPLC grade, and distilled water was obtained by a Milli-Q plus water purification system (Millipore Corp., Bedford, MA). Edoxin (cyclophosphamide injection) was procured from Apollo Pharmacy.

### Plant material

The tender leaves of *C. papaya* were collected in July 2012 from Jamia Hamdard Campus. The leaves were authenticated from NISCAIR by taxonomist Dr. H. B. Singh (NISCAIR/RHMD/Consult/-2012-13/2158/164).

### Quality control analysis of extract

#### Sample preparation

The fresh leaves were cleaned of the debris matter, depetiolated after removing woody stalks, and washed with purified water. The cleaned leaves (300 g) were manually chopped and crushed directly for one min in a grinder with the addition of small amount of distilled water. The volume of mixture obtained was made up to 1000 mL with the addition of distilled water and kept at ambient temperature for 24 h with occasional shaking. It was filtered off using muslin cloth and the marc left was washed with distilled water. The filtrate and washings were pooled together and the final volume was made up to 1000 mL. The collected juice/extract was subjected to freeze drying and stored at −20 °C until further use. The freeze-dried powder was subjected to quantitative estimation of phytoconstituents for standardization and quality control prior to use for biological activity.

#### Quantitative estimation of myricetin, caffeic acid, *trans*-ferulic acid, and kaempferol using validated HPTLC method

HPTLC method was developed for standardization of freeze-dried powder in the light of simultaneous quantitative estimation of some important phenolic compounds and flavonoids including myricetin, caffeic acid, *trans*-ferulic acid, and kaempferol. Chromatography was performed on 20 × 10 cm aluminium HPTLC plates coated with 0.2 μm layers of silica gel 60F_254_. Samples were applied as bands of 4.0 mm wide, 8.3 mm apart by the use of a CAMAG (Switzerland) Linomat V sample applicator fitted with a microliter syringe. A constant application rate of 120 nL/s was used. Linear ascending development, to a distance of 80 mm, with toluene:ethyl acetate:formic acid (50:40:10; v/v/v) as mobile phase, was performed in a 20 × 10 cm twin-trough glass chamber, saturated with mobile phase for 15 min. The developed plates were air-dried and scanned at 320 nm by CAMAG TLC densitometric scanner III operated by winCATS software. The source of radiation was a tungsten lamp, the slit dimensions were kept at 4.0 × 0.3 mm, and the scanning speed was 10 mm/s. The method was validated as per the ICH guidelines for calibration, linearity, precision, accuracy, robustness, specificity, LOD, and LOQ as per previously described methods (Singh et al. 2011; Tiwari et al. 2012; Parveen et al. 2014; Ahmad et al. 2015). The analysis was carried out in triplicate.

#### Ultra-performance liquid chromatography–mass spectrometry (UPLC-qTOF/MS) analysis

The LC-MS was performed on Water’s ACQUITY UPLC^TM^ system (Serial No # F09 UPB 920 M; Model code # UPB; Waters Corp., Milford, MA) equipped with a binary solvent delivery system, an auto sampler, column manager, and a tunable MS detector (Serial No # JAA 272; Synapt; Waters, Manchester, UK). Chromatographic separation was performed on a Water’s ACQUITY UPLC^TM^ BEH C18 (100.0 × 2.1 mm ×1.7 μm) column at 40 ± 5 °C. The mobile phase used for UPLC analysis consisted of 0.5% formic acid (A) and acetonitrile (B), at a pH of 2.7, which was degassed before analysis, and the gradient elution was 2 to 100% (B) in 40 min at a flow rate of 0.5 mL/min and 25 °C. The injection volume was 20 μL. The column and auto sampler were maintained at 40 ± 5 °C and 25 ± 5 °C, respectively, and the pressure of the system was set to 15000 psi.

#### qTOF/MS conditions

The mass spectrometry was performed on a quadruple orthogonal acceleration time of flight tandem mass spectrometer (Waters Q-TOF Premier^TM^, Micromass MS Technologies, Manchester, UK). The nebulizer gas was set to 500 L/h, the cone gas was set to 50 L/h, and the source temperature was set to 100 °C. The capillary voltage was set to 3.0 kV and the sample cone voltage was set to 40 kV. Argon was employed as the collision gas at a pressure of 5.3 × 10^−5 ^Torr. The Q-TOF Premier^TM^ was operated in V mode with resolution over 8500 mass with 1.0 min scan time and 0.02 sec interscan delay. The accurate mass and composition for the precursor ions and for the fragment ions were calculated using MassLynx v 4.1 software incorporated with the instrument.

#### In vitro antioxidant activity and quantitative estimation of total flavonoid and phenolic content

The total flavonoid (Ahmad et al. 2014) and phenolic contents were determined, and the 2,2-diphenyl-1-picrylhydrazyl (DPPH) radical scavenging activity (Singh et al. 2015) as well as β-carotene bleaching assay (Li et al. 2009) in SCPLE was carried out.

### Antithrombocytopenic activity

#### Animals and dosing schedule

Female albino Wistar rats (200–300 g) were procured from Central Animal House Facility, Jamia Hamdard, New Delhi. The animals were divided into four groups of six each. Group I and group II served as normal and toxic controls, respectively. Groups II–IV received CP (25 mg/kg, i.p.) for first three days, consecutively. Groups III and IV served as treatment groups receiving, SCPLE, 50 and 150 mg/kg b.wt, p.o., respectively, from day 1 to day 14. In the absence of any treatment, both groups I and II received 0.8 mL normal saline, orally. First treatment with CP was counted as day one. One hour after last treatment, blood samples were collected from each animal and subjected to Coulter Counter for analyses of platelet count (PC), total leucocyte count (TLC), differential leucocyte count (DLC) including neutrophils, lymphocyte, monocyte, eosinophil, basophil, bleeding time, and clotting time (Duke 1910). The dose of drug extract was calculated from the human dose (10–20 g of papaya leaf, oral), as mentioned in the Ayurvedic Pharmacopoeia of India (API 2008) using the method described by Reagan-Shaw et al. (2008). A total of five blood collections were performed in all the animals on day 0, 3, 7, 11, and 15 of the experiment after administration of SCPLE. The experimental protocol was approved by Institutional Animal Ethics Committee (IAEC) with registration number 173/CPCSEA/937. The animals were kept in polypropylene cages under standard laboratory conditions (25 ± 2 °C; photoperiod of 12 h) and had a free access to commercial pellet diet and tap water *ad libitum*. Rats were killed by cervical dislocation, and liver and spleen of animals were isolated to study any histopathological changes occurred due to various treatments.

### Immunomodulatory activity

#### Animals

Fifty-four female Swiss albino mice, weighing 30–45 g, procured from Central Animal House, Jamia Hamdard, New Delhi. The animals were divided into nine groups of six each and maintained as per the standard protocol of CPCSEA guidelines. Group I served as normal control received normal saline (0.2 mL), groups II–IX received CP (50 mg/kg body weight, i.p.) consecutively for three days on 1st, 2nd, and 3rd day of experiment, and groups IV–IX received CP (50 mg/kg body weight, i.p.) on day 8, 9, and 10. Groups II, IV, VI, and VII served as toxic control, whereas groups III, V, VII, and IX served as treatment and received SCPLE (150 mg/kg/day p.o.). The animals of groups II and III were immunized by injecting 0.1 mL of 1 × 10^8^ cells of fresh sheep red blood cells (SRBC) suspension, i.p, on 6th day for humoral antibody and delayed-type hypersensitivity response.

#### Humoral immune response (HA) and delayed-type hypersensitivity (DTH)

Blood samples were collected in microcentrifuge tubes from animals of groups I, II, and III on 10th day and serum was separated. Antibody levels were determined by haemagglutination technique (Bin-Hafeez et al. 2001). The delayed-type hypersensitivity response was determined by the reported method (Bin-Hafeez et al. 2003). Further, animals were again challenged with 0.1 mL of 1 × 10^8^ SRBC in the left hind footpad and the right footpad with the same volume (0.1 mL) of normal saline. The thickness of the footpad was measured using digital vernier caliper after SRBC administration at 0, 24, and 48 h. The pre- and postchallenge difference in the thickness of footpad was expressed in mm and taken as a measure of delayed-type hypersensitivity (Bin-Hafeez et al. 2001).

#### Assessment of nonspecific immune response

##### Effect on pro-inflammatory cytokine production in cyclophosphamide-treated animals

Animals of groups IV and V were used for this study as toxic control and treatment, respectively. The six hours after last dose of CP, the blood was collected to separate serum. It was used for analysis of cytotoxicity using specific quantitative rat TNF-α ELISA kit (Manu and Kuttan 2009).

##### Cyclophosphamide-induced neutropenia

In this study, animals of groups VI and VII were used as toxic control and treatment, respectively. These groups received neutropenic dose of CP on last day (200 mg/kg, s.c.), instead of 50 mg/kg i.p. The blood was collected for TLC prior to and after 3rd day of injection of neutropenic dose of CP (Thatte et al. 1987).

##### *In vivo* carbon clearance assay

Finally, the groups I, VII, and IX were used to carry out *in vivo* carbon clearance assay. All the groups received Indian ink dispersion (0.1 mL/30 g body weight, i.v.) on 10th day of study. The blood was collected at 2-min and 10-min intervals after injection of ink dispersion. The sodium carbonate solution (4.0 mL, 0.1% w/v) was added to the blood samples to lyse the erythrocytes. Absorbance was taken at 675 nm using spectrophotometer. The mean of phagocytic index was calculated for each group of animals (Gokhale et al. 2003).

### Statistical analysis

The values were expressed as mean ± SEM. Statistical analyses were performed using one-way analysis of variance (ANOVA) followed by Student’s *t*-test for antithrombocytopenic activity and Dunnett’s *t*-test for immunomodulatory activity. *p* ≤ 0.05 was considered as significant. All statistical analyses were performed using the GraphPad software (San Diego, CA).

## Results

The FDP yielded 4.843% w/w of fresh juice, which was used for HPTLC analysis and UPLC-qTOF/MS fingerprinting for quality control as well as for determination of antioxidant, antithrombocytopenic, and immunomodulator activity.

### Quantitative estimation of myricetin, caffeic acid, *trans*-ferulic acid, and kaempferol using validated HPTLC method

**Figure 2.**
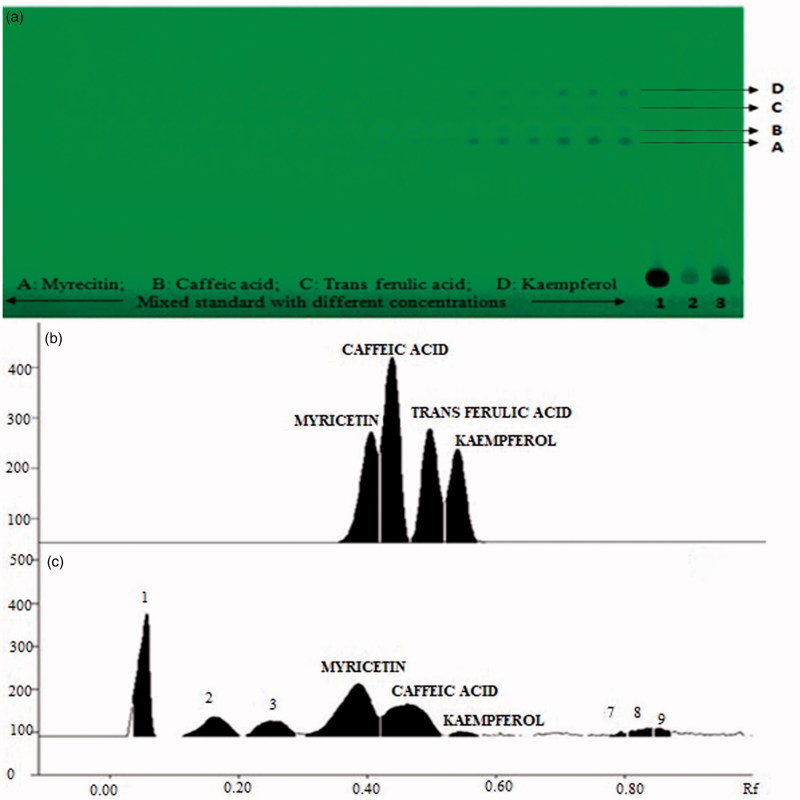
Developed HPTLC plate showing spots of myricetin, *trans*-ferulic acid, caffeic acid, and kaempferol at different concentrations in standards and in samples (1: mother extract) at 254 nm (A), HPTLC chromatogram of sample showing peaks of myricetin, *trans*-ferulic acid, caffeic acid, and kaempferol (B).

The scanning of developed plates at 320 nm produced well-separated peaks of myricetin, caffeic acid, *trans*-ferulic acid, and kaempferol at R_f_ value 0.39 ± 0.01, 0.44 ± 0.02, 0.50 ± 0.01, and 0.55 ± 0.01, respectively (Figure 2). The developed simultaneous estimation method was found linear in the wide range of 50–1250 ng with good regression coefficient (0.99 ± 0.001), and LOD and LOQ were calculated to be in range of 3.50–18.76 ng/spot and 10.63–56.87 ng/spot (Table 1). The content of myricetin, caffeic acid, *trans*-ferulic acid, and kaempferol was analyzed, which showed 280.16 ± 5.99, 370.18 ± 6.27, 1110.86 ± 2.97, and 160.53 ± 2.48 (μg/g) in CPL mother extract. The interday, intraday, and interlaboratory precision at three concentration levels (50, 500, and 1000 ng/spot), in terms of %RSD, was in the range of 0.05–2.00 for all the compounds. The low values of %RSD obtained after incorporating small but thoughtful changes in wavelength (Figure 3(A(i,ii))) and in composition of solvent system indicated robustness of the methods (Figure 3(B(i,ii))). The specificity of the method was determined by analyzing standard drugs and samples. The accuracy of the developed method calculated by spiking 0, 50, 100, and 150% of analyte in pre-analyzed samples showed recovery in the range of 96.83–102.76% of all markers (Table 1).

Figure 3.(A(i)) Robustness of the HPTLC method for estimation of mixed standards by changing detecting wavelengths (*n* = 3); (ii) robustness of the HPTLC method for estimation of mixed standards by changing detecting wavelengths (*n* = 3) showing Levy-Jennings Plot of %RSD for markers; (B(i)) robustness of the HPTLC method for estimation of mixed standards by changing detection of mobile phase composition (*n* = 3); (ii) robustness of the HPTLC method for estimation of mixed standards by changing detecting of mobile phase composition (*n* = 3) showing Levy-Jennings plot of %RSD for markers.

**Figure:**
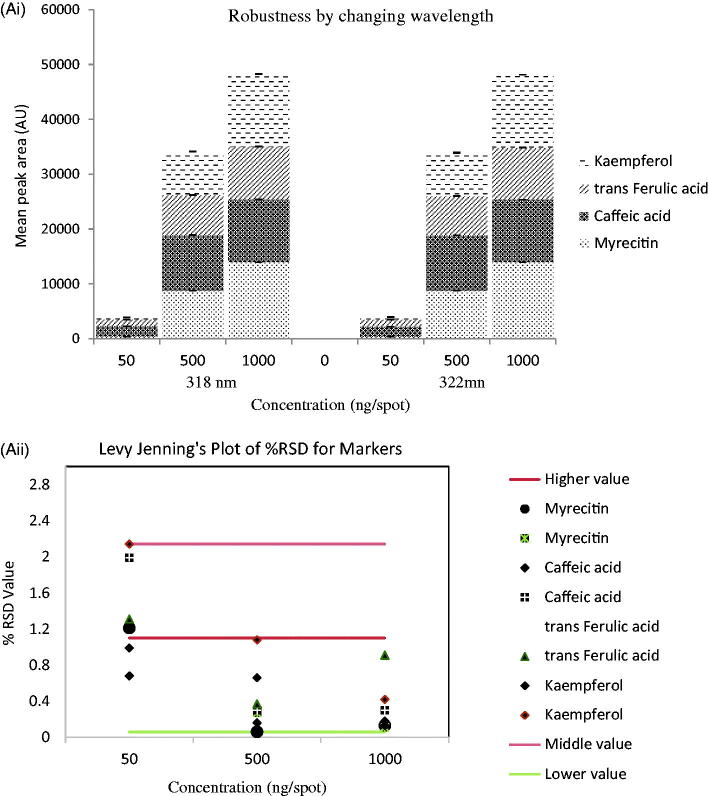

**Figure:**
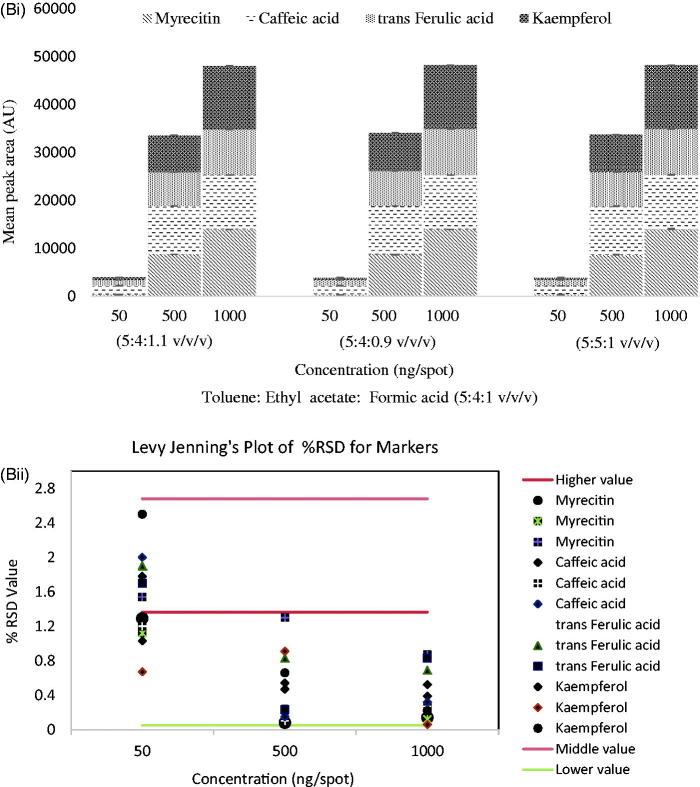

**Table 1.** Linearity and validation data of chromatographic HPTLC method for mixed standards (*n* = 3).

	Biomarkers			
Parameters	Myricetin	Kaempferol	*Trans*-ferulic acid	Caffeic acid
Linearity range (ng/spot)	25–1250	25–1250	25–1250	50–1250
Regression equation	Y= 17.98 x ± (−283.5)	Y= 15.82x ± (−248.4)	Y= 11.58 x ± 1359	Y= 15.26x ± 2465
Regression coefficient ± SD	0.997 ± 0.001	0.998 ± 0.001	0.993 ± 0.002	0.997 ± 0.001
Slope ± SD	17.96 ± 0.015	15.82 ± 0.005	11.56 ± 0.015	15.25 ± 0.025
Intercept ± SD	283.50 ± 0.100	248.43 ± 0.057	1358.60 ± 0.577	2465 ± 0.577
LOD (ng/spot)	04.502	03.508	05.129	18.767
LOQ (ng/spot)	13.643	10.632	15.544	56.872
Precision (%RSD range)				
Interday	0.87–1.98	0.67–1.83	0.29–2.00	0.44–1.75
Intraday	0.05–1.08	0.12–1.52	0.83–1.90	0.36–1.82
Interlaboratory	0.12–1.50	0.50–1.93	0.48–1.47	0.35–0.82
Accuracy (% drug recovered)	99.90–100.08	99.61–100.35	98.30–102.76	96.83–101.43

### UPLC-qTOF/MS fingerprint of SCPLE

**Figure 4.**
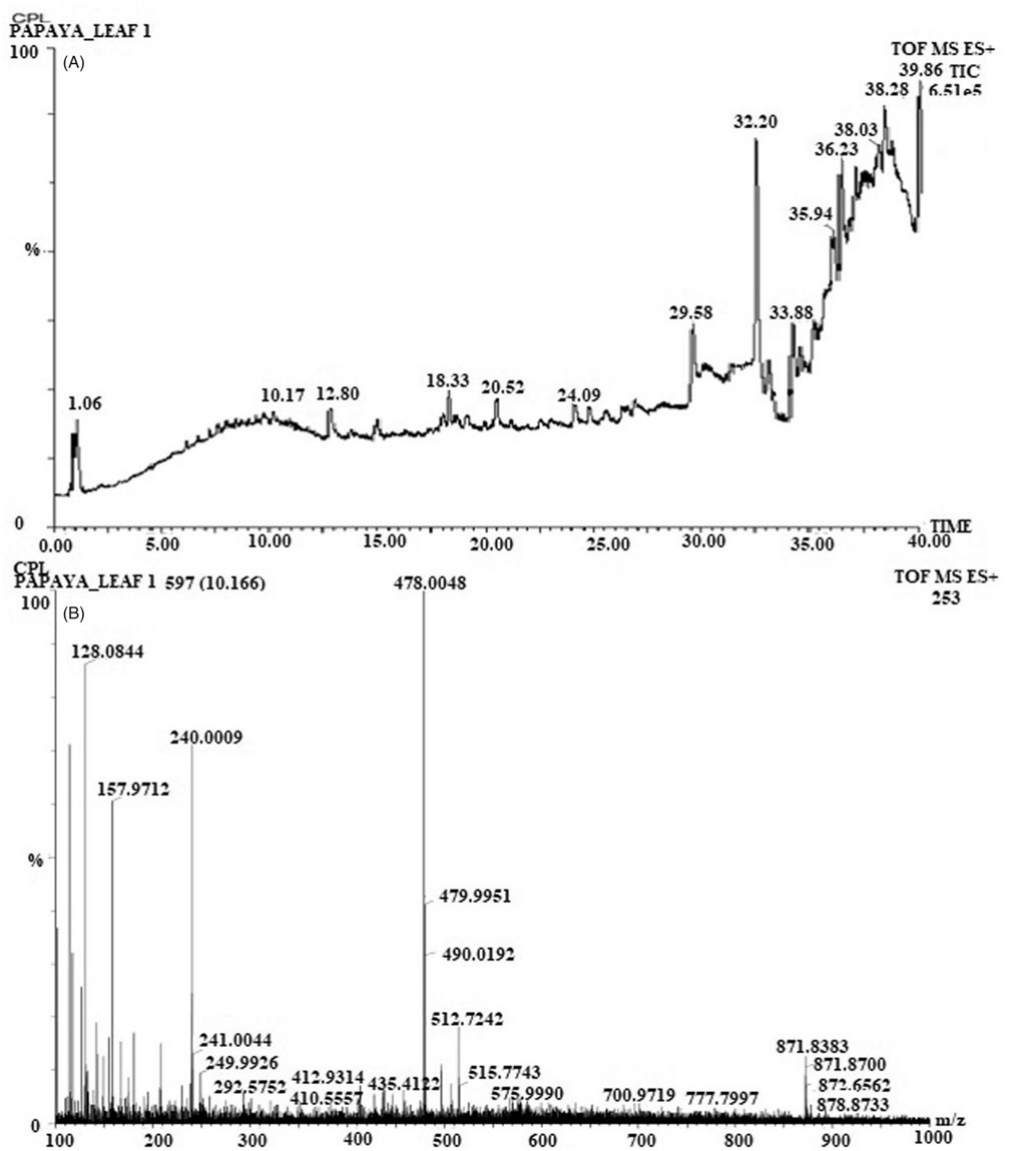
UPLC chromatogram of *C. papaya* leaf (A), mass spectra of *C. papaya* leaf aqueous extract at 320 nm in ESI^+^ mode (B).

**Figure 5.**
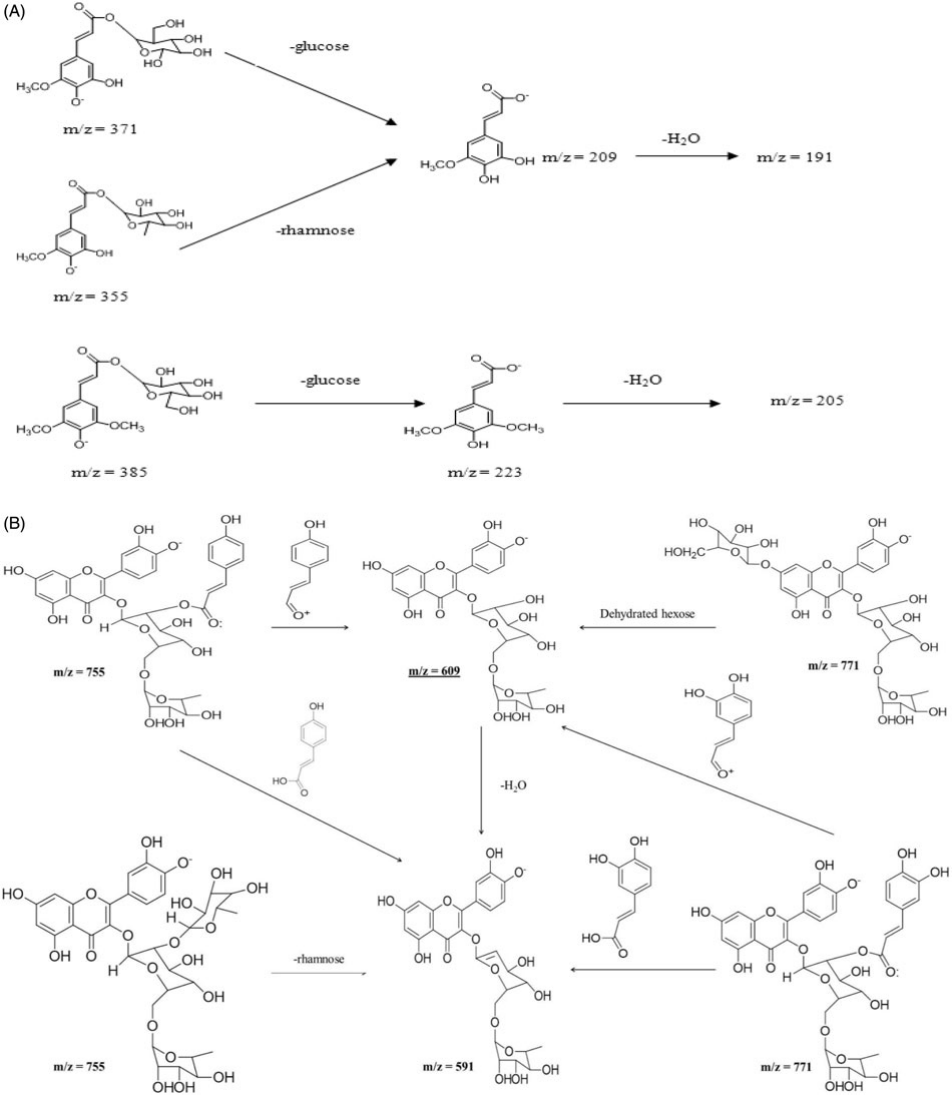
Proposed fragmentation of cinnamic acid derivatives (A) and quercetin derivatives (B) tentatively identified in papaya leaf aqueous extract.

In order to identify components present in the samples, qualitative analysis was carried out on an UPLC coupled to ESI quadrupole time-of-flight mass spectrometry (UPLC-ESI-qTOF/MS) in ESI^+^ mode. The potential calculated masses and elemental compositions associated with the measured mass of compounds were generated and studied with MassLynx (Figure 4(A)). The retention time obtained for 22 peaks were at 1.05, 6.71, 7.67, 8.43, 9.76, 10.17, 10.93, 12.80, 15.01, 18.00, 18.33, 20.52, 23.09, 24.09, 24.73, 26.93, 28.20, 29.58, 32.20, 36.23, 38.28, and 39.86 min. Furthermore, the UPLC with ESI/MS was used to generate a phenolic profile of SCPLE. Figure 4(B) shows mass spectra of SCPLE recorded at 320 nm; Figure 1 shows some tentatively identified chemical structures, whereas Figure 5(A,B) shows the proposed fragmentation of cinnamic acid and quercetin derivatives. A total of 25 compounds detected in UPLC-qTOF/MS out of which one was unknown, and six phenolics, two alkaloids, and sixteen were hydroxycinnamic acid derivatives and flavonoids (Table 2).

**Table 2.** Tentatively identified phenolics and flavonoids in *C. papaya* leaf aqueous extract by UPLC-qTOF/MS.

	[M-H] (*m/z*)	[M-H] (m/z)	
Rt (min)	Online	Reported	Tentative identification
1.05	–	262	Unknown
1.05	371	370	Hydroxyl-ferulic acid-hexoside
6.71	415	415	β-Sitosterol
7.67	516	519	Isochlorogenic acid
7.67	625	629	Quercetin-3-*O*-(2′ hexosyl)-hexoside (Q-3-*O*-sophoroside)
8.43	610	608	Quercetin-3-*O*-rutinoside (rutin)
8.43	784	787	Sinapic acid-hexoside
9.76	133	130	Malic acid
10.17	478	479	Carpaine
10.93	130	130	2-Keto-hexanoic acid
12.80	168	167	Vanillic acid
15.01	130	130	3-Keto-*n*-caproic acid
18.00	371	370	Hydroxyl-ferulic acid-hexoside
18.33	355	355	Coumarylglucaric acid
20.52	166	167	*p*-Coumaric acid
23.09	194	200	Ferulic acid
24.09	258	256	Carpamic acid
24.73	222	221	Ferulic acid ethyl ester
26.93	148	149	*trans*-cinnamic acid
28.20	286	282	Kaempferol
29.58	280	280	Linoleic acid
32.20	282	282	*Trans*-2-Oleic acid
36.23	282	282	3-Octadecylenic acid
38.28	338	338	Quercetin
39.86	282	282	4-Octadecylenic acid

### *In vitro* antioxidant activity and quantitative estimation of total flavonoid and phenolic content

The metabolite analysis data showed that total flavonoid content was found to be 16.74 ± 0.50 QE/g in SCPLE, whereas total phenolic content was 29.54 ± 0.02 mg GAE/g. CPL exhibited dose-dependent inhibition of DPPH radical in a stoichiometric manner. The IC_50_ values of the butylated hydroxy anisole, butylated hydroxy toluene, ascorbic acid, and SCPLE were 2.03 ± 0.19, 0.95 ± 0.30, 4.17 ± 0.02, and 4.93 ± 0.98 μg/mL, respectively. The β-carotene content in 100 g of SCPLE was found to be 779.69 ± 5.55 μg.

### *In vivo* antithrombocytopenic evaluation

**Figure 6.**
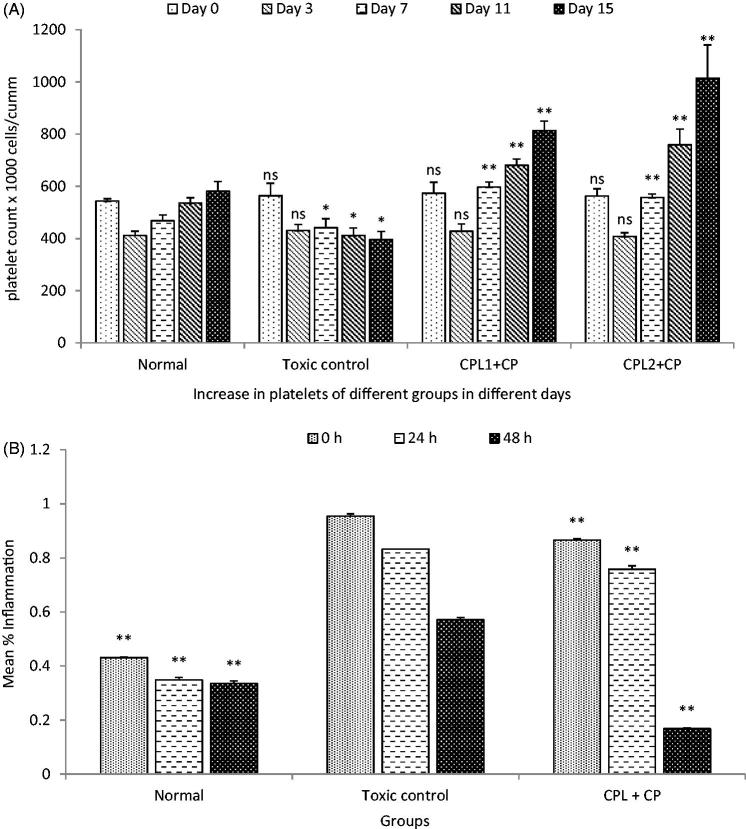
(A) Effect on blood for platelet count against cyclophosphamide after oral administration of SCPLE; (B) effect of aqueous extract of *C. papaya* leaf on delayed-type hypersensitivity. (All values are expressed as mean ± SEM (*n* = 6)). *** indicates *p* < 0.001 extremely significant, ** indicates *p* < 0.01 highly significant, * indicates *p* < 0.05 significant, and ns indicates *p* > 0.05 nonsignificant when compared with toxic control group where GP: group; CYP: cyclophosphamide; CPL1:*C. papaya* leaf (dose 50 mg/kg); CPL2: *C. papaya* leaf (150 mg/kg).

**Figure 7.**
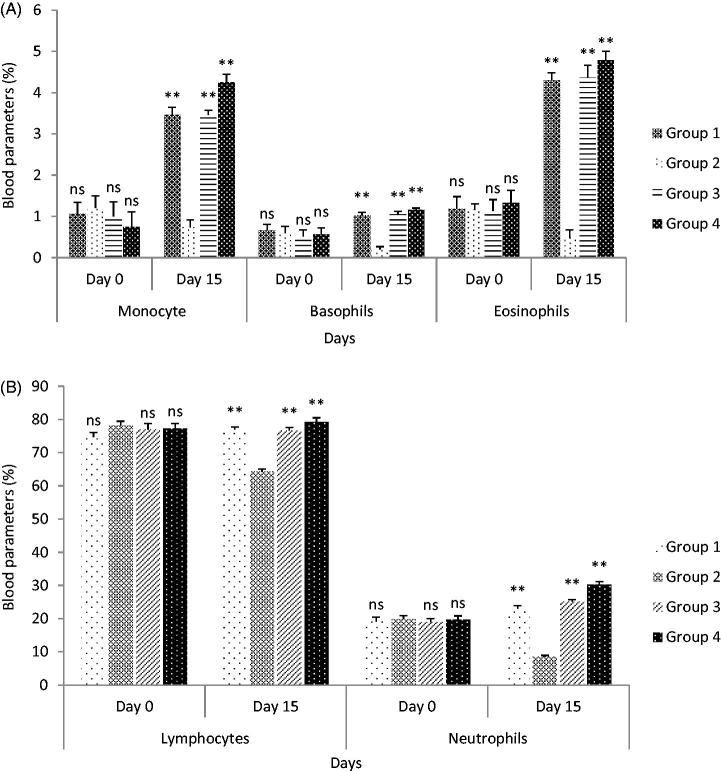
(A) Effect of standardized aqueous plant extract and cyclophosphamide on monocyte, basophils, and eosinophils; (B) effect of standardized aqueous plant extract and cyclophosphamide on lymphocyte and neutrophils. All values are expressed as mean ± SEM (*n* = 6). *** indicates *p* < 0.001 extremely significant, ** indicates *p* < 0.01 highly significant, * indicates *p* < 0.05 significant, and ns indicates *p* > 0.05 nonsignificant when compared with toxic control group, where group 1: normal; group 2: negative control receiving cyclophosphamide; group 3: treatment group receiving *C. papaya* leaf (dose 50 mg/kg); group 4: treatment group receiving *C. papaya* leaf (150 mg/kg).

The present study examined the platelet increasing effect on oral administration of SCPLE on oral administration in both normal and thrombocytopenic rats. It was observed that PC, TLC, and neutrophils decreased from normal level. On day 3rd, PC was 408.5 × 10^3^ cells/mm^3^, TLC 7.88 × 10^3^ cells/mm^3^, and neutrophils 68%, while after administration of SCPLE on day 15, the PC was 1014.83 × 10^3^ cells/mm^3^, TLC 12.90 × 10^3^ cells/mm^3^, and neutrophils 85% (Figures 6(A) and 7(A,B)). The SCPLE caused significant increase in all the indices related to PC, TLC, and DLC in group IV (*p* < 0.01).

**Figure 8.**
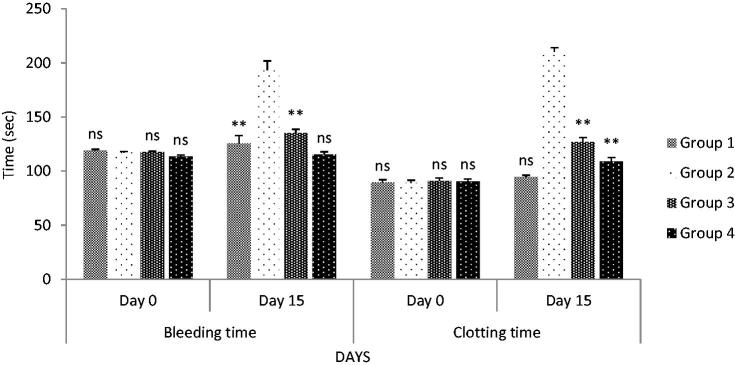
Effect on bleeding time and clotting time after oral administration. All values are expressed as mean ± SEM (*n* = 6). *** indicates *p* < 0.001 extremely significant, ** indicates *p* < 0.01 highly significant, * indicates *p* < 0.05 significant, and ns indicates *p* > 0.05 nonsignificant when compared with toxic control group, where group 1: normal; group 2: negative control receiving cyclophosphamide; group 3: treatment group receiving *C. papaya* leaf (dose 50 mg/kg); group 4: treatment group receiving *C. papaya* leaf (150 mg/kg).

The bleeding time and clotting time were significantly (*p* < 0.05) lowered after oral administration of SCPLE in groups III and IV, as compared to toxic control (Figure 8). However, better results were shown by group IV as compared to group III receiving 50 mg/kg, body weight.

### Histopathological studies

**Figure 9.**
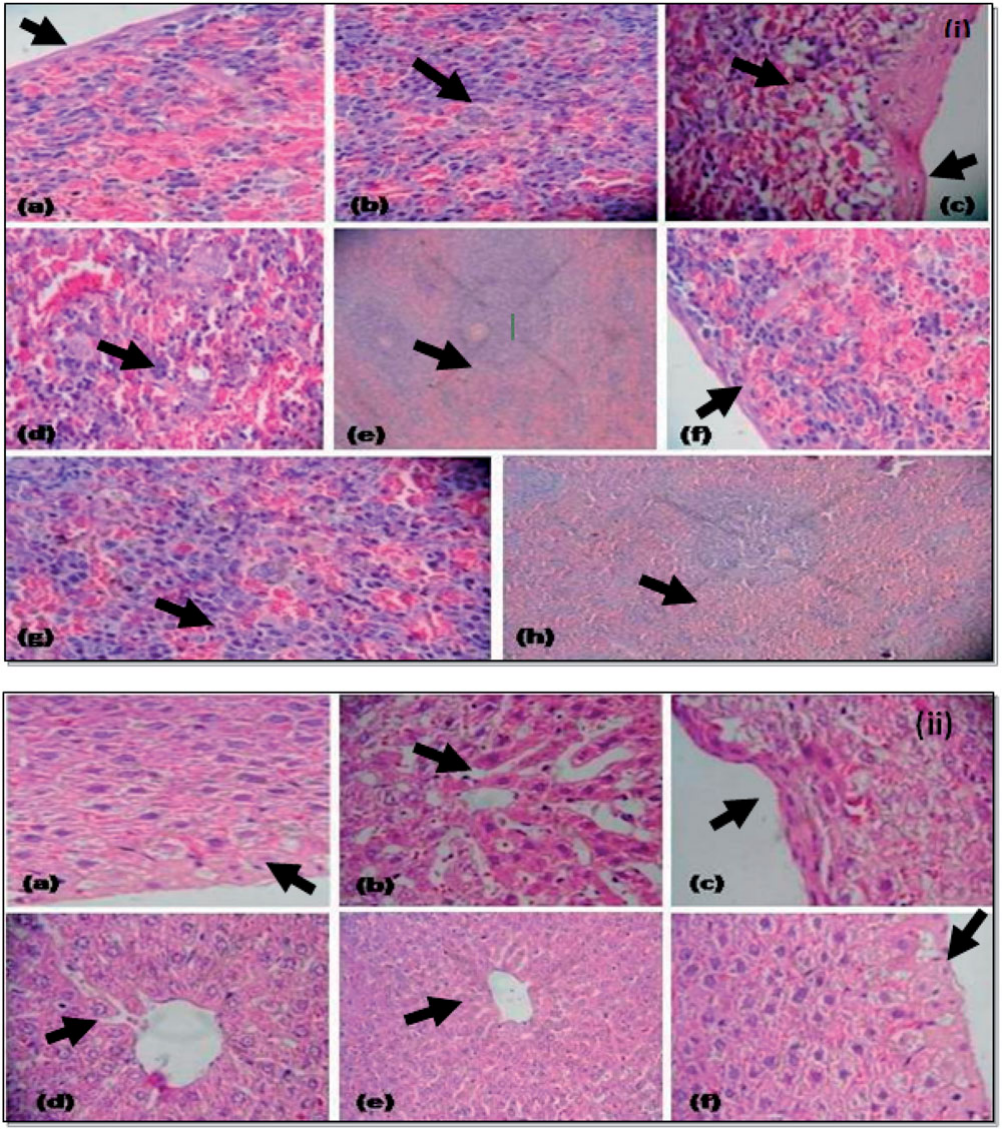
(i) Histopathology of spleen: (a) histology of spleen from normal group showing normal fibrosis thickening; (b) normal clarification of cells; (c) toxic control group showing fibrosis capsular thickening and increase in cytoplasmic vacuolation at focal places; (d) decreased EMH release; (e) increased cellularity in white pulp; (f) treatment group showing minimal or no cytoplasmic vacuolation at focal places with less fibrosis thickening; (g) enlarged cell size at focal places and maximum EMH release; (h) normal cellularity in white pulp; (ii) histopathology of liver: (a) histology of liver from normal control showing normal cell size, hepatocytes, less cytoplasmic vacuolation, normal fibrosis thickening, cytocidal space is also less; (b) histology from toxic control (thrombocytopenic) group showing large sinusoidal space; (c) degeneration of cytoplast and maximal fibrosis capsular thickening; (d) maximum density of cells; (e) histology from treatment group showing maximal EMH release; (f) degeneration of cytoplast and scattered fibrosis capsular thickening.

Examination of liver sections of thrombocytopenic animals showed inflammation of hepatocytes with increased sinusoidal space along with capsular fibrosis; however, minimal effect was observed by SCPLE-treated group. There is minimal or no cytoplasmic vacuolation and cytocidal space observed at focal places. The cell size was enlarged in the treatment groups, when compared to normal and toxic groups (Figure 9(i)). Evidence of sinusoidal dilation was also found in the spleen of animals in treatment groups. The SCPLE showed minimal sinusoidal space, degeneration of cytoplast, scattered fibrosis capsular thickening, and maximal EMH release, as compared to toxic and normal groups (Figure 9(ii)).

### Immunomodulatory activity

The CP showed significant inhibition in antibody titre response, while SCPLE showed an elevated response in terms of rank of cups of titre plate, similar to normal group (*p* < 0.01) (Table 3). Significant increase in DTH response was observed as an increased mean paw oedema value (*p* < 0.01) in extract-treated groups, when compared with toxicant group receiving CP (Figure 6(B)).

**Table 3.** Effect of *C. papaya* leaf on total leucocyte count, *in vivo* carbon clearance test and cyclophosphamide-induced neutropenia, haemagglutination antibody titre, and pro-inflammatory cytokine production in cyclophosphamide-treated animals.

Parameters	Group 1 Normal	Group 2 Negative control	Group 3 Treatment
TLC (×10^3^) ± SEM	10350 ± 178.42**	5450 ± 296.37	11408.33 ± 392.13**
Phagocytic index (Mean ± SEM)	0.013 ± 0.0010**	0.032 ± 0.0005	0.025 ± 0.0006**
Induce neutropenia (cells/cm) ± SEM	6600 ± 301.11**	2201.66 ± 45.491	2566.66 ± 8.819^ns^
Mean antibody titre (Mean ± SEM)	0.33 ± 0.009**	0.57 ± 0.009	0.16 ± 0.006**
TNF-α (Mean ± SEM)	24.1 ± 0.9*	646.53 ± 23.99	131.99 ± 17.39*

All values are expressed as mean ± SEM (*n* = 6). Dunnett’s test was used for statistical significance assessment.

*** indicates *p* < 0.001 extremely significant.

** indicates *p* < 0.01 highly significant.

* indicates *p* < 0.05 significant; ns indicates *p* > 0.05 nonsignificant when compared with toxicant where group 1: normal; group 2: negative control receiving cyclophosphamide; group 3: treatment group receiving *C. papaya* leaf (150 mg/kg).

The neutropenic dose (200 mg/kg, s.c.) of CP significantly reduced the TLC in control animals (*p* < 0.01). Co-administration of SCPLE produced reversal of TLC in treatment groups. The neutrophil count (%) was highly reduced in CP-treated toxic control, when compared to initial values on day ‘0’. The CP-induced neutropenia model is dependent on the effect of drugs on haematopoietic system. The SCPLE also showed good carbon clearance test and high phagocytic index (*p* < 0.001). The SCPLE has significantly inhibited the production of TNF-α in CP-treated animals as shown in Table 3.

## Discussion

The papaya leaves are rich source of α-tocopherol, ascorbic acid, flavonoids, phenolics, cyanogenic glycosides, and glucosinolates (Singhai et al. 2016) and have been used since ages in dengue fever (Dharmarathna et al. 2013; Zunjar et al. 2016) that is why the leaf extract was selected for antithrombocytopenic and immunomodulatory activity.

Various compositions of mobile phases were tested to get better resolutions of myricetin, *trans*-ferulic acid, caffeic acid, and kaempferol. The resolution of all components with symmetrical and reproducible peaks was achieved by using mobile phase consisting of toluene: ethyl acetate: formic acid (50:40:10, v/v/v) at R_f_ 0.39 ± 0.01, 0.44 ± 0.02, 0.50 ± 0.01, and 0.55 ± 0.01, respectively. The CPL aqueous extract showed the presence of myricetin, *trans*-ferulic acid, caffeic acid, and kaempferol using HPTLC. Comparison of the UV spectral characteristics of all peaks for the standards revealed the identity of same peaks in the sample. The calibration curves were linear in the range of 25–1250 ng for myricetin, *trans*-ferulic acid, caffeic acid, and kaempferol. The *trans*-ferulic acid was present in higher amount (1110.86 ± 2.97 ng/g) of all the active ingredients that were recorded in CPL aqueous extract, which are in corroboration with earlier report (Singhai et al. 2016).

The qualitative analysis of various phenolics and flavonoids by UPLC-qTOF/MS in SCPLE resulted in tentative identification of various metabolites. A total of 25 compounds detected in UPLC-qTOF/MS, out of which 24 were identified tentatively. Among them, six were phenolics, two alkaloids, and 16 hydroxycinnamic acid derivatives. The metabolite analysis of SCPLE showed high level of flavonoids and phenolic content. The data showed that total flavonoids and total phenolic content were higher as compared to previous reports (Vuonga et al. 2013). The platelet-enhancing effect of SCPLE showed good results from the present study; however, it does not directly pinpoint to an exact mechanism of action. In normal healthy conditions, the platelets are produced from megakaryocytes within 4–6 days (Moreau et al. 2016). Nevertheless in our study, a dramatic increase in platelets was observed within 72 h. It is plausible to hypothesize about the platelet increasing effect of the SCPLE, since it may be either due to megakaryopoietic/thrombopoietic stimulatory activity and/or induced due to splenic contractions. A synergistic effect of these two mechanisms may also operate. The enhanced bleeding time predisposes to prolonged haemorrhage and consequently excessive blood loss. This directly correlates with the decrease in number of platelets in circulation (Subenthiran et al. 2013). Improvement of bleeding time is critical in haemorrhagic disorders, such as dengue haemorrhagic fever to reduce the number and severity of bleeding episodes. This may be partly due to stimulatory effect of SCPLE on the lymphocytes and bone marrow haematopoietic cells (Haque and Ansari 2014). The clotting time measures the degree of activation of coagulation pathways and the functionality of the clotting factors.

Tissue examination of the liver and spleen provided further evidence of extramedullary haematopoiesis as induced by CP. The liver damage leads to an increase in blood pressure of the portal veins, which impede the flow of blood out of spleen leading to sequestering of platelets in spleen (Maan et al. 2015). The dilation of sinusoids of above organs results in poor venous outflow. The white pulp was observed in T cells and B cells, which were located to provide an immune response to blood borne antigens. The red pulp serves as to filter blood constituents and to store RBC and thrombocytes (Dennison and Farrell 2015). Spleen hyperplasia and congestions are the results of liver damage.

The plant products (such as phenolics, organic acids, flavonoids, and glucosinolates), being used as immunomodulator in the indigenous system, have been reported to modulate the immune system in various *in vivo* models (Izquierdo-Vega et al. 2017). The SCPLE improved humoral immunity as shown in the indirect haemagglutination test, also the cell-mediated immunity and carbon clearance significantly increased but there was a reduction in CP-induced neutropenia (Nagarathna et al. 2014). The indirect haemagglutination test was performed to confirm the effect of SCPLE on the humoral arm of immune system. The humoral immunity involves interaction of B cells with the antigen and their subsequent proliferation followed by differentiation into antibody-secreting plasma cells. Antibody binds to antigen and neutralizes its elimination by cross-linking to form clusters that are more readily ingested by phagocytic cells (Srinivasan et al. 2016). This indicates that the enhanced responsiveness of macrophages and T and B lymphocyte subsets involved in the antibody synthesis (Savant et al. 2014). The DTH is antigen specific, which causes erythema and induction at the site of antigen infection in immunized animals. The DTH can be different for different species, but the general characteristics like influx of immune cells at the site of injection, macrophages, and basophils in mice become apparent within 24–72 h. T-cells are required to initiate the reaction (Yuk et al. 2017). Increase in the DTH response indicates that the drug has stimulatory effect on lymphocytes and/or other necessary cell types required for the expression reaction (Haque and Ansari 2014). The DTH requires the specific recognition of a given antigen by activated T lymphocytes, which subsequently proliferate and release cytokines. This results in increase in the vascular permeability; induces vasodilatation, macrophage accumulation, and activation; and promotes the phagocytic activity.

The increase in the carbon clearance index reflects the enhancement of the phagocytic function of mononuclear macrophage and nonspecific immunity by the cells of reticuloendothelial system (RES). The RES is a diffuse system consisting of phagocytic cells, which play an important role in the clearance of particles from blood stream. When colloidal carbon particles in the form of ink are injected directly into the systemic circulation, the rate of clearance of carbon from blood by macrophage is governed by an exponential equation (Sharma and Rangari 2016). Since SCPLE augmented the circulating antibody titre, it was worthwhile for evaluating its effect on peripheral blood count and CP-induced immunosuppression. The CP-induced neutropenia model concentrates on the effect of drugs on the haematopoietic system (Fu et al. 2015). Tumour necrosis factor-alpha (TNF-α) is a pro-inflammatory cytokine that mediates through inflammatory response in the body, whereas interleukin-1 (IL-1) is a cytokine with diverse immunologic, physiologic, and haematopoietic effects. Through a series of biological events, IL-1β increases the expression of TNF-α receptors in peripheral blood (Tian et al. 2014). The reversal of hypersensitivity, neutropenia, phagocytic function, and pro-inflammatory cytokine production by treatment with SCPLE may be because of the presence of antioxidant phytoconstituents like phenolics, flavonoids, and carotenoids and as evident from the DPPH assay.

The results of present investigation clearly indicate that SCPLE produces increase in the PC and fall in the various biochemical parameters namely bleeding time and clotting time because of the formation of platelet plug at the time of excessive bleeding, whereas increase in TLC and DLC associated with increased thrombocytes without possessing toxic effect on any organ. Hence, SCPLE can be used for a longer time for diseased conditions, where PC drastically decreases and it is also used to boost the body’s immunity.

## Conclusions

The developed HPTLC was used for simultaneous quantitative analysis of phenolic acids and flavonoids in SCPLE. UPLC-qTOF/MS method was developed for qualitative analysis of six phenolics, two alkaloids, sixteen hydroxycinnamic acid derivatives, and flavonoids to develop metabolite signature of SCPLE. The results of *in vivo* studies proved usefulness of SCPLE in thrombocytopenia, which was evidently confirmed by histological studies of spleen and liver followed by assessment of immunomodulatory activity. The oral administration of 150 mg/kg body weight of SCPLE caused significant increase in PC, TLC, and DLC with reduced bleeding time and clotting time without any acute toxicity. The proposed study therefore provides a great future of SCPLE for dengue fever-related thrombocytopenia in the era of phytopharmaceuticals.
